# From Bright Ideas to Tools: The Case of Malaria

**DOI:** 10.1371/journal.pntd.0003377

**Published:** 2015-01-08

**Authors:** Melba F. Gomes, Annette C. Kuesel

**Affiliations:** Special Programme on Research and Training in Tropical Diseases (TDR), a co-sponsored Programme of UNICEF/UNDP/World Bank/WHO, based at the World Health Organization, Geneva, Switzerland; RTI International, United States of America

## Introduction

Securing the right of the world's poor to live and thrive by developing effective weapons to prevent, reduce, cure, or eliminate infectious diseases was the goal underpinning the creation of the United Nations Children's Fund (UNICEF)/United Nations Development Program (UNDP)/World Bank/World Health Organization (WHO) Special Programme on Research and Training in Tropical Diseases (TDR) [1]. At the time of its creation, 1975, the WHO Smallpox Eradication Unit had successfully led, and was on the verge of concluding, smallpox eradication efforts [2]. Hope was high that a targeted tropical disease program could bring state-of-the-art knowledge to the development of new tools to reduce the large burden of six diseases—malaria, schistosomiasis, trypanosomiasis, leishmaniasis, filariasis, and leprosy [3]. Tool development required knowledge, and knowledge required research.

**Figure 1:**
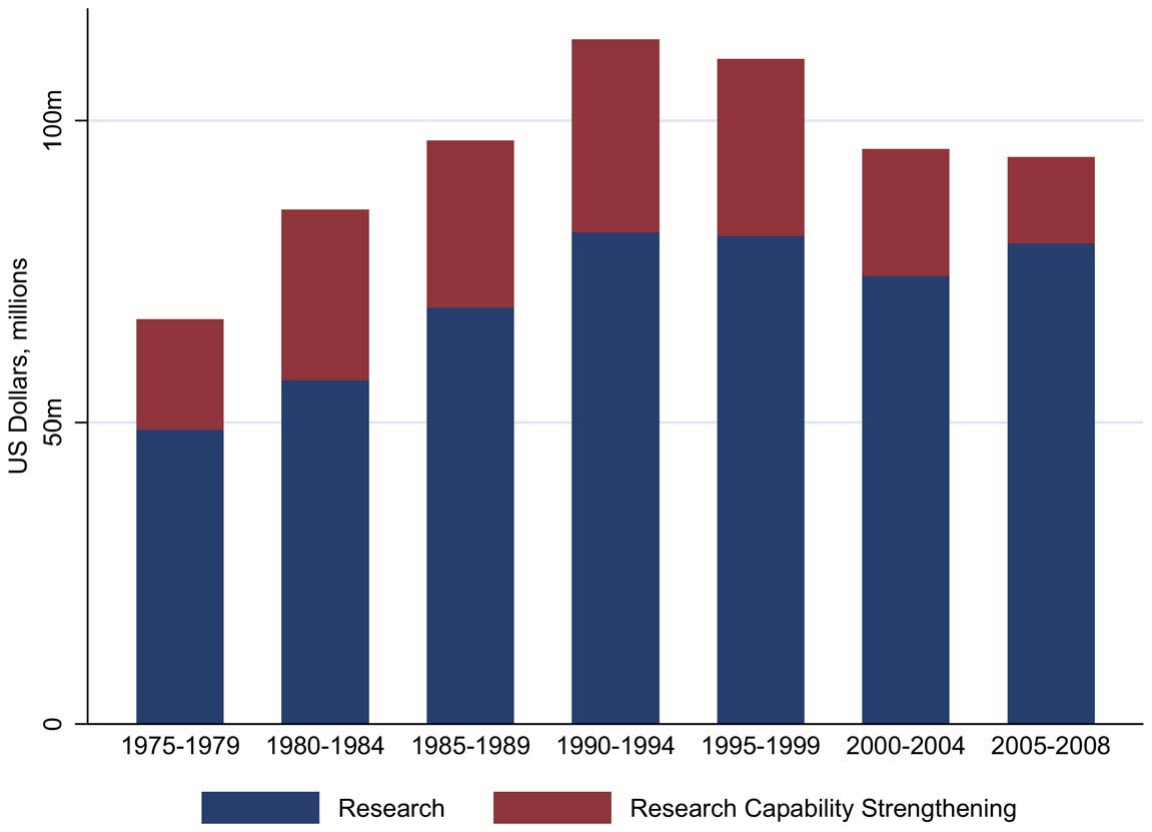
TDR Research and Research Capacity Strengthening (RCS) funding, 1975–2008.

The best science was clearly the place to start. Scientific Steering committees were created to fund the best scientific ideas in each disease, to upgrade research capacity to self-sufficiency in disease-endemic countries (through a Research Capacity Strengthening Committee [RCS]) and to improve the delivery of new tools and understand economic aspects of disease control (through a Social and Economic Research Committee [SER]). These committees reviewed and funded research annually or biannually, assessing the best ideas, whatever their origin, much like the “Grand Challenges” approach of today. Scientific peer reviews regularly fine-tuned the structure and direction of research undertaken and approved budgetary allocations. The exception was RCS, which received 25% of the Programme budget until around 2004 (Fig. 1), thus safeguarding one of TDR's goals—to develop local capacity to contribute research for disease control [4].

The budget allocated to research (excluding program costs) was above US$20 million annually, with the largest contributions from Scandinavian countries; the United States; and the UNDP, World Bank, and WHO as co-sponsors (1974–1992) [5] and thereafter from increasingly diverse designated funders [6]–[7].

Throughout, TDR kept internal electronic records of the research it funded, until 2008, when the records management system changed for the whole of the World Health Organization. In this paper, we use TDR internal data from 1975 to 2008 to review changes in strategy and funding which separated the first 20 years from subsequent years, focusing on malaria. We provide a personal perspective and some reflections on the rationale underpinning the changes.

## The “Grand Challenges” Approach of the First Two Decades

In 1975, there was limited knowledge about effective malaria control. The main measures were drugs (chloroquine) for treatment of infected patients and propoxur plus sulfalene pyrimethamine for spraying mosquito resting places. But these approaches, even when used consistently and together, had difficulty in controlling malaria in high-transmission areas [8].

TDR began by supporting basic science: at the time it was assumed that solutions would derive from better understanding of how the various parasite stages function in humans, how the host responds and acquires immunity, and from vector biology. Drugs could be developed if research could identify molecules necessary for parasite survival or target parasite proteins that help it bind to host blood cells for nutrition or mediate host responses to invasion. Knowing how immunity works to limit parasite density and clinical response would advance blood stage vaccine development. Inducing host immunity against sexual parasite stages might block transmission. Vector biology research would isolate targets for new insecticides and establish how to block or genetically modify mosquito transmission potential. Thus, it was expected that basic knowledge about cellular interactions would drive drugs, vaccines, and vector control solutions; where, when, and in which population tools should be deployed would be informed by epidemiological evidence.

These assumptions dictated the functional architecture of TDR malaria research in the Programme's first 20 years. Three malaria committees presided: CHEMAL—Chemotherapy of malaria, IMMAL—Immunology of malaria, and FIELDMAL—Epidemiology of malaria. Each committee reviewed and funded research proposals received according to scientific merit, design, feasibility, and budget relative to funds available. Good ideas thrived.

The strategy changed with the results of a FIELDMAL-funded randomized controlled trial in The Gambia which showed a 70% malaria-specific and 63% all-cause mortality reduction when young children (at highest risk from malaria) were protected by insecticide impregnated (treated) bednets while they slept [9]. These results were spectacular and warranted immediate action. The urgent question was whether, and to what extent, impregnated bednets could reliably save children from dying in other African areas with different (longer or more intense) malaria seasons. The Programme decided to answer this important question reliably and decisively. It fully financed and coordinated three major trials in different African epidemiological conditions: Burkina Faso [10], Ghana [11], and Kenya [12], plus an implementation program in The Gambia [13]; the United States Agency for International Development (USAID) funded an additional trial in a hyperendemic area of Kenya [14], [15].

By 2003, strong evidence was available that impregnated bednets could reduce mortality by 17% compared to no nets, and later trials examining morbidity showed protection from illness as well. However it could be predicted that bednet use and scale-up would not be easy—there was no clear health system structure to drive use. This was an intervention at the household level and community involvement, commitment, and understanding, along with changes in traditional ways of thinking about malaria control—long dependent upon drugs and insecticide spraying—would be required. Some scientists were also worried about prevention of the natural development of immunity to malaria through widescale use of impregnated bednets, and potential for postponing death to later ages as a result [16].

The consequences, and irony, of the early bednet results reverberated through the Programme. Set up to develop tools from the best science, the Programme had proven that a major solution for childhood malaria mortality reduction in Africa had been obtained through use of a simple, low-tech, practical, physical impediment between mosquitoes and humans that killed the former and reduced death in the latter.

The strategy of the Steering Committee approach—funding promising ideas—led to the first trial. But it had taken a concentrated, targeted approach to demonstrate impact. The latter strategy and the speed of its success was driven by the Secretariat.

## Strategic Changes

There were four consequences to the way in which TDR worked and to its interactions with scientists and control programmes: (1) total malaria funding increased over the next decade, (2) regional distribution of funding shifted to Africa, (3) TDR's architecture and approach changed, and (4) TDR became far more involved in the process of bringing evidence to policy.

### Increase in malaria field research

Between 1977 and 1993, malaria funding by the three malaria Steering Committees of TDR averaged US$3 million–US$4 million per biennium per committee. Thus, for many years, the TDR budget for malaria activities was 38% (approximately US$9 million) of the total budget (including malaria research funded by RCS). By the time the mega-bednet trials were ongoing, malaria research rose to almost 50% of TDR spending, the highest proportion of the total TDR budget dedicated to the disease [5]. Financial allocations to other malaria components of the Programme were unaffected since the increase towards malaria research was covered by additional funding support. Although the risk taken by the Programme in concentrating so much effort on the bednet trials was unprecedented, the return on the investment turned out to be extremely high when mortality reduction was confirmed in all epidemiological situations [17].

### Shifting funds to the location of the problem

**Figure 2:**
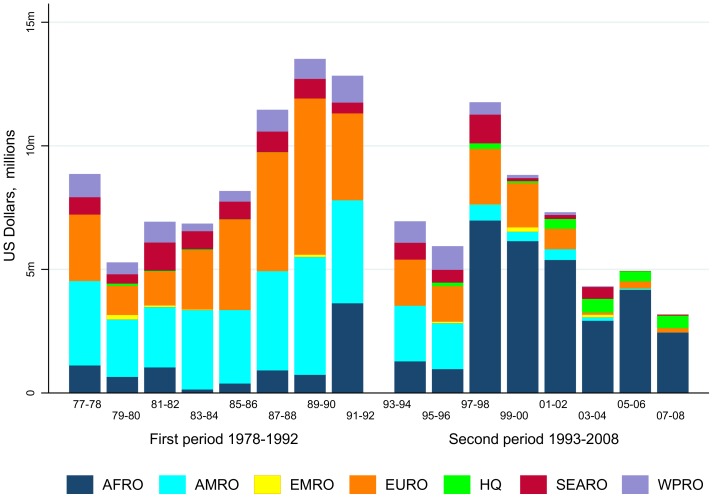
Funding of malaria research by WHO region: 1977–1992 versus 1993–2008. AFRO, WHO region for Africa; AMRO, WHO region for the Americas; EMRO, WHO region for Eastern Mediterranean; EURO, WHO region for Europe; HQ, WHO Headquarters; SEARO, WHO region for South-East Asia; WPR, WHO region for the Western Pacific.

The success of the new approach of taking a single good idea to large-scale implementation in disease-endemic countries had financial consequences. An analysis of TDR funding by region (Fig. 2) shows a shift of research funds from the US/Americas (AMRO) and Europe (EURO) towards Africa (AFRO), excluding diagnostic research and RCS funding via the Multilateral Initiative on Malaria (MIM) [4]. Resources which had hitherto been spent on good ideas in basic research or discovery—and most of these research proposals had come from the developed countries (1978–1992)—rapidly shifted after 1993 to research conducted in malaria-endemic countries of Africa. Thus, TDR research funds began to move to where the problem was—towards locations and institutions in Africa capable of implementing large-scale clinical and field trials.

### Architectural changes and goal-oriented approaches

**Figure 3:**
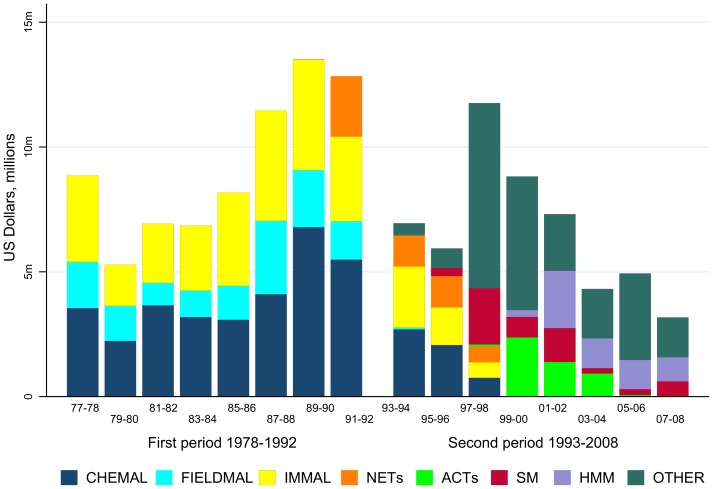
Funding of malaria research by activity: committees 1977–1992 versus task forces 1993–2008. CHEMAL, Chemotherapy of malaria; FIELDMAL, Epidemiology of malaria; IMMAL, Immunology of malaria; NETs, bednets; ACTs, artemisinin combination therapy; SM, severe malaria; HMM, home management of malaria.

Internally, there was a shift from the “responsive” mode, in which allocation of funds was determined by direct competition between proposals submitted by investigators, towards a combined approach that included “targeted” funding for very specific goals. Task forces were created, each with an important question to answer, such as reducing drug resistance or determining whether early treatment of uncomplicated or severe malaria reduced mortality. Projects submitted for funding to the task forces were selected based on merit and direct or indirect relevance to the overall goal of the task force (Fig. 3).

The Programme began to institutionalize the powerful strategy used experimentally for impregnated bednets—which had authoritatively demonstrated the efficiency of supporting a line of inquiry to conclusion. A task force on bednets (NETs) succeeded FIELDMAL, and this task force funded multidisciplinary research to overcome operational obstacles to successful implementation. Basic and vector research addressed challenges of new insecticides, insecticide-resistance, and long-lasting insecticide-impregnated bednets. Operational research was funded to take the results into practice—demonstrating feasibility, providing financial evidence for policy decisions, implementing health systems research, and assessing community mobilization options.

Many African countries did not have a critical mass of local researchers to support operational research, and it had not been lost on the Programme leadership that the bednet mortality trials had depended substantially on expatriate scientists in African malaria-endemic stations [18]. Consequently, the Programme included capacity building, mentorship, career re-entry grants, and master's and PhD support in funding through the new task forces, linked to the RCS Unit [4].

The change towards task forces was partly motivated by the realization that different approaches were important. A new applied field research unit was set up to house the task forces. Twenty years from its inception, the pace of antimalarial drug resistance was increasing while industry's involvement in drug development for tropical diseases was shrinking, with many companies handing over leads to TDR for exploitation [3]. At the same time (1991–1995) only about 5% of global research and development (R&D) investment was directed to reducing the tropical disease burden [19] and the Programme was troubled about future prospects [18]. A product development unit was created to support the development and registration of drugs, including antimalarials [20].

### New tools for malaria control

In chemotherapy of malaria, to the disappointment of many, the CHEMAL Committee was discontinued, but several practical areas of therapeutic research came under task forces. With the artemisinins becoming the only option for therapy for severe malaria in South East Asia, mechanisms for protection against artemisinin resistance were taken up by the task force on resistance/artemisinin combination therapy (ACTs). This task force funded a series of studies demonstrating the value of artemisinin combination therapy to delay drug resistance to the artemisinins, which (until then) were available only as monotherapy [21]–[23], and demonstrated superiority of ACTs over existing treatments. The trials were largely (but not entirely) funded by TDR, and simultaneously revealed that the comparator drugs being used for treatment (chloroquine and sulphadoxine pyrimethamine) totally or largely lacked efficacy. By implication, low efficacy was likely responsible for an excess of deaths that could be prevented by switching to ACTs as soon as possible. These scientific results eventually changed both policy and practice [24].

The task force on severe malaria (SM) and home management of malaria (HMM) turned the spotlight to the community. The severe malaria task force funded the initial trials on Intermittent preventive treatment in infants (IPTi), the Tanzania results of which [25] led to a consortium exploring and showing the benefit of the approach in different countries in Africa [26]. This task force also funded research on rectal artesunate showing superiority of rectal artesunate to quinine, which led to the decision to register rectal artesunate for the initial management of severe malaria when parenteral treatment is not available [27], [28]. Eventually it funded the largest randomized, controlled study of severe malaria and provided the evidence for use of a single dose of rectal artesunate to reduce death and permanent disability in patients unable to get to clinics quickly [29].

The home management of malaria task force was set up to improve febrile case management in children close to the home, following clear results from Ethiopia that effective community case management saved lives [30]. This approach later became the basis for community-based research in support of Integrated Childhood Case Management (iCCM) [31], [32]. Task force funding was supplemented by funding support to other malaria research projects submitted to TDR, as reflected in Fig. 3.

**Figure 4:**
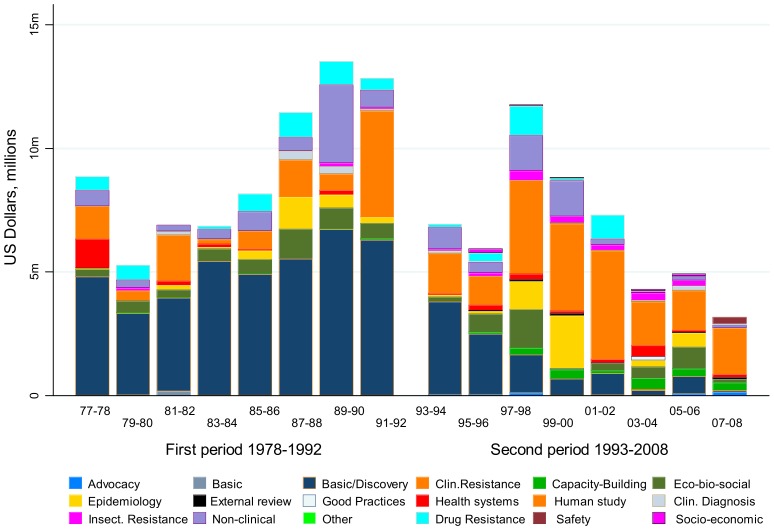
Variety of disciplines involved in malaria research funded: committees 1977–1992 versus task forces 1993–2008.

Fig. 4 illustrates the limited number of disciplinary areas covered by malaria committees versus the wider range of disciplines funded by malaria task forces set up to achieve more targeted goals. Although it is obvious that the malaria task forces were largely clinically oriented in nature, they also covered a variety of operational research and implementation issues that were fundamental to a broader understanding of how best to control malaria and increase access to essential medicines.

## Policy Impact

The evidence from TDR's malaria research funding from 1993 onwards—impregnated bednets, ACTs, IPTi, rectal artesunate, iCCM—has contributed to WHO Malaria Treatment Guidelines [33] and continues to form the backbone of malaria control efforts today [34]. In addition to the individual life-saving benefits of effective protection and treatment, some of these strategies were later shown to exert a population effect: consistent and widespread use of impregnated bednets in a geographical area protected others in the community by reducing transmission [35], while the switch to ACTs has conferred the same benefit through superior gametocytocidal effects [36], [37].

All of these interventions took simple ideas to fruition. By the time The Global Fund for HIV, Tuberculosis (TB), and Malaria was set up in 2002, countries could seek financial support to increase access to several tools to control malaria, most developed at TDR, thus substantially contributing to the reductions in malaria mortality observed in the past decade.

## Reflections

Most of this success and impact on policy was due to TDR's ability to anticipate future needs and act fast. Moreover, its use of randomized, multicountry trials for answering important questions in malaria increased the reliability of the evidence and its relevance to different populations.

TDR was challenged often. At a time when the standard approach was facility care, the Programme's emphasis on access to timely, even presumptive, management of febrile illness in the community went against traditional facility-based case management. It funded cluster-randomized and individually randomized trials, sometimes with designs that were controversial [29]. In predicting eventual resistance to the artemisinins, it tested approaches to increase the lifespan of these compounds and, together with the Global Malaria Programme, advocated strongly for first-line use of such combination therapy to save lives, despite increased cost to donors and control programs [24].

In hindsight, it is remarkable how much was accomplished with a limited budget in the decade between 1993 and 2003 and how simple and obvious some of the ideas tested now appear. Major solutions for mortality reduction in malaria had not come from profound, powerful, cellular knowledge of host–parasite–vector interactions or from identifying antigens and enzymes as targets for vaccines and drugs, but from testing simple solutions derived from observation of what might work—from physical barriers between mosquitoes and humans to practical rectal and oral formulations.

The real innovation was not to wait for high-tech solutions, but to focus on the need and take some ideas to conclusion. TDR lost time thereafter but appears to be set on course again [38], [39].
